# Alzheimer’s Disease: In Vitro and In Vivo Evidence of Activation of the Plasma Bradykinin-Forming Cascade and Implications for Therapy

**DOI:** 10.3390/cells13242039

**Published:** 2024-12-10

**Authors:** Allen P. Kaplan, Berhane Ghebrehiwet, Kusumam Joseph

**Affiliations:** 1 Department of Medicine, The Medical University of South Carolina, Charleston, SC 29425, USA; 2 Department of Medicine, Stony Brook University, Stony Brook, NY 11794, USA; berhane.ghebrehiwet@stonybrookmedicine.edu; 3 Astria Pharmaceuticals, Boston, MA 02210, USA; kjoseph@astriatx.com

**Keywords:** Alzheimer’s, bradykinin, kallikrein

## Abstract

The plaques associated with Alzheimer’s disease are formed as a result of the aggregation of Aβ peptides, which vary in length from 38 to 43 amino acids. The 1-40 peptide is the most abundant, while the 1-42 peptide appears to be the most destructive to neurons and/or glial cells in a variety of assays. We have demonstrated that aggregated Aβ, a state prior to plaque formation, will activate the plasma bradykinin-forming pathway when tested in vitro. Aggregation is zinc-dependent, optimal at 25–50 µM, and the rate of aggregation is paralleled by the rate of activation of the bradykinin-forming pathway as assessed by plasma kallikrein formation. The aggregation of Aβ 1-38, 1-40, and 1-42 is optimal after incubation for 3 days, 3 h, and under 1 min, respectively. The cascade is initiated by the autoactivation of factor XII upon binding to aggregated Aβ; then, prekallikrein is converted to kallikrein, which cleaves high-molecular-weight kininogen (HK) to release bradykinin. Studies by a variety of other researchers have demonstrated the presence of each “activation-step” in either the plasma or spinal fluid of patients with Alzheimer’s disease, including activated factor XII, kallikrein, and bradykinin itself. There is also evidence that activation is more prominent as dementia worsens. We now have medications that can block each step of the bradykinin-forming pathway as currently employed for the therapy of hereditary angioedema. Given the current state of therapy for Alzheimer’s disease, which includes monoclonal antibodies that retard the rate of progression by 30% at most and have significant side effects, it seems imperative to explore prophylaxis using one of the long-acting agents that target plasma kallikrein or factor XIIa. There is a long-acting bradykinin antagonist in development, and techniques to target kallikrein mRNA to lower levels or knock out the prekallikrein gene are being developed.

## 1. Introduction

Alzheimer’s disease is one of the devastating disorders that have an association with aging; there are also familial forms that tend to begin earlier, which, together, have a major impact on health worldwide. Therapy is limited in terms of choices, most importantly, no treatment has a large or consistent effect on disease progression. These include cholinesterase inhibitors such as Donepezil, inhibitors of N-methyl D-aspartate such as memantine, and three monoclonal antibodies directed to the Aβ protein constituent of the plaques that are deposited in key regions of the brain. The first, aducanumab, has modest efficacy, and its approval by the US FDA was considered controversial. The next two, lecanemab and donanemab, may have somewhat greater efficacy, up to a 30% reduction in the rate of disease progression; however, cerebral micro-hemorrhage, as a possible side effect, is a limiting consideration regarding their use. It is clear that any new approach that has a chance of slowing or possibly arresting disease progression would be a welcome advance. Every possibility with a good scientific basis should be pursued.

In this manuscript, we will review the published data on the ability of aggregated Aβ protein to activate the plasma bradykinin-forming cascade. This in vitro phenomenon is proportional to the extent of aggregation, a predecessor of plaque formation, and is dependent on the zinc ion concentration. In addition, studies of patients with Alzheimer’s disease demonstrate the same activation steps leading to bradykinin formation, namely, the formation of two enzymes, activated factor XII and plasma kallikrein. The extent of activation appears proportional to the extent of memory loss in patients. The ability to block this cascade with medication is available; thus, we can test the hypothesis that this inhibition would have a positive effect on the course of the disease.

## 2. Activation of the Bradykinin-Forming Cascade by Aggregated Aβ

The Aβ protein deposits extracellular plaques within cerebral blood vessels and brain parenchyma [1,2], a characteristic abnormality in brain biopsies of patients with Alzheimer’s disease. The protein responsible for plaque formation is heterogeneous and varies in length from 38 to 43 amino acids; however, the 38, 39, and 43 length amino acid proteins are present in small amounts, while the 40 and 42 amino acid moieties appear to be far more active when tested for effects on inflammation [3,4]. The 40 amino acid peptide is the most abundant, while the 42 amino acid peptide appears to be particularly damaging to neurons [5].

**Figure 1:**
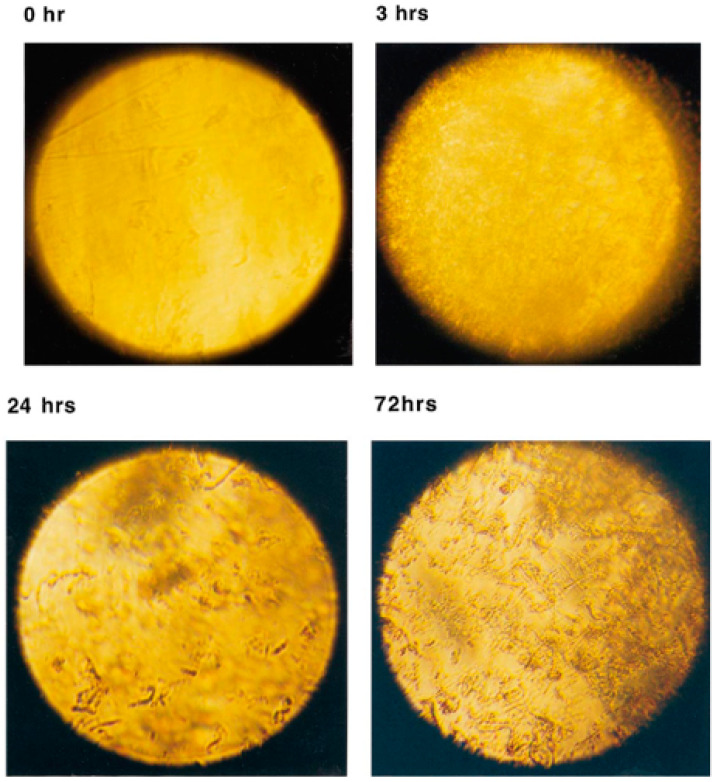
Time-dependent aggregation of Aβ (1-40) at 500 µg/mL in Hepes buffer with 0.02% NaN3 was incubated in a 96-well microtiter plate with a tight lid for 72 h at 37 °C. At the indicated time, aggregation of Aβ (1-40) was visualized with an Olympus CK2 optical inverted microscope [6].

We have assessed the ability of the Aβ protein to activate the plasma bradykinin-forming pathway and found that the aggregation of the protein is an absolute requirement [6] and aggregation is dependent on zinc ion [6]. A depiction of the time dependence of aggregation is shown in Figure 1, in which the aggregation of Aβ (1-40) was visualized with an Olympus CK2 optical inverted microscope. A comparison at 8 h, 24 h, and 72 h using a starting solution of 500 mg/mL is shown. A variety of aggregated proteins have been shown to initiate bradykinin formation [7], a somewhat surprising finding since typical non-organic activators are highly negatively charged, e.g., glass, silicates, celite, and uric acid crystals [8], while some physiologic activators are highly negatively charged as well, e.g., mast cell heparin [9,10] or platelet polyphosphate. However, this is not true of cryoproteins (aggregated immunoglobulin) or the aggregated Aβ. If the “typical” pathway is activated, the initiator binds to factor XII, augments intrinsic (but trace) activity within the native protein [11], and initiates autoactivation (cleavage at Arg–val) [12] to form activated factor XII, which, in turn, converts plasma prekallikrein to the active enzyme kallikrein [13]. Kallikrein digests a high-molecular-weight form of kininogen (HK) at two loci; it cleaves a lys-arg bond and an arg-ser bond to release the amino acid peptide, bradykinin. With arg at the N-terminus and C-terminus, bradykinin is exceedingly “basic” and acts at bradykinin B-2 receptors (constitutively present on vascular endothelial cells), causing the vessels to dilate with a concomitant and prominent increase in vascular permeability. Manifestations differ for arterial vessels vs. venous vasculature since the latter is associated with the leakage responsible for angioedema [14] while the former can cause hypotension or shock [15]. Its role in brain physiology is not yet clear.

Our initial studies employed the activation of normal plasma or the activation of the cascade using purified proteins. The Aβ was purchased from Sigma (St. Louis, MA, USA) and included peptides that were 1-38, 1-40, and 1-42 amino acids long. In our study, the aggregation of the 1-38 protein continued to increase over a period of days, and we empirically used material pre-incubated for 3 days in the studies of bradykinin generation. With the 1-40 peptide, we achieved the same effect with a 3 h preincubation, so this appeared to be most useful, particularly for kinetic experiments. The 1-42 protein was fully aggregated in less than a minute; in fact, it was significantly aggregated even after a few seconds and was difficult to employ for experiments in which the timing of the various incubations was critical.

**Figure 2:**
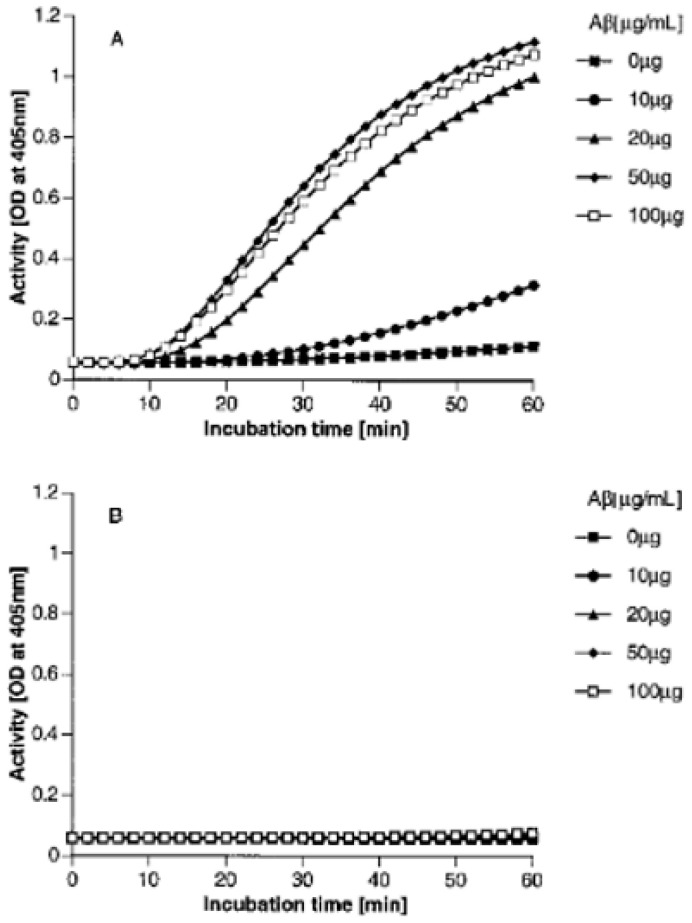
Aggregated Aβ (1-38) induces the FXII-dependent conversion of prekallikrein to kallikrein in the presence of zinc ion. Human Aβ (1-38) at 500 µg/mL was preincubated for 3 days at 37 °C to form aggregates. Purified human proteins, FXII, HK, and prekallikrein, each at 1 µg/mL, were incubated with 0.6 mM chromozym-PK, a synthetic substrate for plasma kallikrein, and with 0, 10, 20, 50, or 100 µg/mL Aβ (1-38), in APMSF-treated Hepes buffer in the presence (**A**) or absence (**B**) of 50 µM zinc ion. This experiment was performed five times with virtually superimposable results; a representative dose response is shown [6].

Figure 2 demonstrates the results from employing 3-day incubated Aβ 1-38 at various concentrations. For studies of aggregation, we prepared a 500 mg/mL stock solution in which the protein was dissolved in 100 µg dehydrated N, N, and dimethylformamide and frozen at −70 °C. It was diluted to the desired concentration with HEPES buffer (mM Hepes, 137 mM NaCl, 11 mM D-glucose, 4 mM KCL, and 1 mg/mL RIA grade bovine serum albumin, pH 7.40, containing 0.02% sodium azide). Purified human proteins factor XII, prekallikrein, and HK, each at 1 µg/mL, were incubated with 0.6 mM chromozyme PK, a synthetic substrate for plasma kallikrein. A time course of Aβ incubation with the three proteins requisite for bradykinin formation is shown in Figure 2A, and an increased rate of kallikrein formation is observed as the concentration of aggregated Aβ is increased. The buffer contained 50 µM zinc. In Figure 2B, the conditions are identical except the zinc was left out. Although zinc does enhance Aβ aggregation, in this instance, we observed that the activation of the proteins required for bradykinin formation by the aggregated Aβ is totally dependent on zinc ion. To examine ion specificity, we determined that calcium, magnesium, or aluminum at concentrations between 10 µM and 2 mM had no effect on activation, while cobalt ion between 15 and 50 µM was as effective as zinc. However, normal plasma zinc is 20–25 µM, within the effective range of zinc ion, while cobalt ion is present at less than 5 µM, below the concentrations where activation is observed.

**Figure 3:**
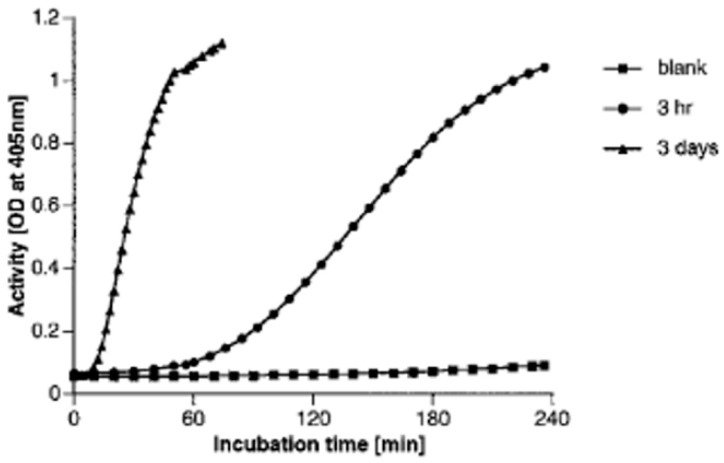
Aggregation of Aβ is required for factor XII-dependent conversion of prekallikrein to kallikrein. Aβ (1-38) at 500 µg/mL was preincubated in Hepes buffer containing 0.02% NaN3 for 3 h or 3 days at 37 °C. The Aβ at 50 µg/mL was then incubated with FXII, HK, and prekallikrein, each at 1 µg/mL, plus 0.6 mM chromozym-PK in APMSF-treated Hepes buffer containing 50 µM zinc ion. Kallikrein activity was assessed at O.D. 405 nm over a 4 h incubation period [6].

The molecule weight of the Aβ monomer is ~4000, a size that would seem unlikely to provide a macromolecular “surface”. Then, we compared the activation by varying the time of pre-incubation of Aβ 1-38 since it aggregates slowly and good kinetics can be obtained. In Figure 3, Aβ 1-38 at 500 mg/mL was incubated for up to 3 days, and we compared samples at the 3 h time point with the 3-day time point, otherwise employing the same conditions as in Figure 1 with zinc at 50 µM. The 3 h incubated Aβ barely activates the system at 1 h, but the activation does increase progressively between 1 and 4 h The 3-day preincubated Aβ significantly activated the kinin-forming protein at 10 min, was more than half-maximal by 30 min, and was close to maximal activation at 60 min.

Next, we wished to examine the requirement for each protein and based the experiments on the known role of each protein in plasma or in a purified protein system. In plasma, prekallikrein circulates bound to HK [16], so interaction with a macromolecular surface such as aggregated Aβ would be expected to involve the direct attachment of factor XII and the binding of the prekallikrein–HK complex via the HK C-terminal end containing domains 5 and 6 [17]. The binding of HK is dependent on the interaction of domain 5 with the initiating surface while domain 6 contains the prekallikrein-binding site [18].

**Figure 4:**
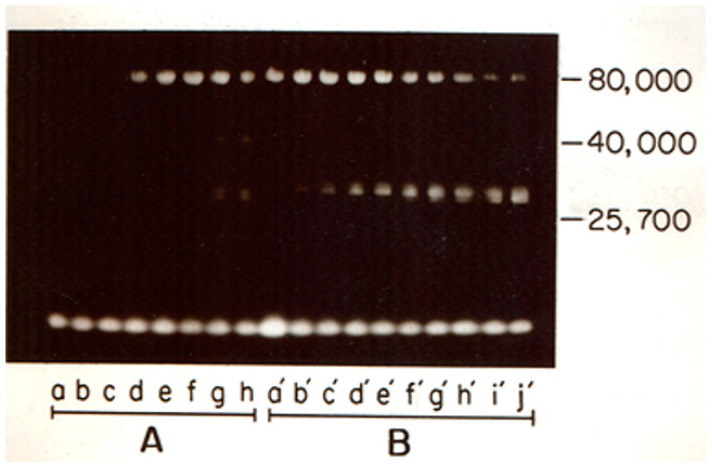
Visualization of the active forms of HF (factor XII) after autoactivation and kallikrein cleavage using the active site inhibitor dansyl-glu-gly-arg chloromethyl ketone (DNS-GGA CMK). Factor XII was incubated with dextran sulfate and was either (A) allowed to be autoactivated or (B) incubated with kallikrein for the following times: (a) 1 min; (b) 5 min; (c) 15 min; (d) 30 min; (e) 45 min; (f) 60 min; (g) 120 min; (h) 180 min; (a′) 1 min, (b′) 3 min; (c′) 5 min; (d′) 10 min; (e′) 15 min; (f′) 30 min; (g′) 45 min; (h′) 60 min; (i′) 120 min; and (j′) 180 min. Aliquots were removed from incubation, treated with DNS-GGACMK, and electrophoresed under nonreducing conditions. Molecular masses are indicated in kilodaltons. The lowest band is unincorporated DNS-GGACMK running at the dye format [21].

The initiation of the cascade is dependent on the activation of factor XII. In this situation, the omission of factor XII eliminates activation. Once a small amount of factor XIIa has formed, prekallikrein is converted to kallikrein, and kallikrein enzymatically converts factor XII to factor XIIa. This “feedback” activation of factor XII [19] is approximately 500-fold faster than the factor XII autoactivation rate [12,20]. Thus, although factor XII serves as the initiator, quantitatively, factor XIIa is mostly a result of activation by kallikrein. Figure 4 demonstrates a time course of the activation of factor XII employing SDS gel electrophoresis activation of factor XII, in which the conversion to the activated forms of factor XII is observed by the activation and cleavage of a flouresceinated synthetic substrate. Factor XIIa (80,000 Kd) appears as the band becomes visible; it is then gradually converted to factor XIIf at ~30,000 Kd.

If this process occurs by the interaction with aggregated Aβ, and prekallikrein is omitted, the rate of factor XII activation would be significantly slower, as is observed in prekallikrein-deficient plasma. This can be directly assayed employing a synthetic substrate cleaved by factor XIIa or a coagulation assay dependent on the factor XIIa activation of factor XI to factor XIa. (It should be clear that in the absence of prekallikrein, no kallikrein can appear and HK will not be cleaved to release bradykinin.) The third protein is HK itself, which, if omitted, precludes any bradykinin from being formed [22,23]. However, both prekallikrein and factor XI circulate bound to HK, and factor XI activation is strongly dependent upon the HK interaction with the initiating surface. Further, the rate of prekallikrein activation by factor XIIa and the feedback activation of factor XII by kallikrein [24] are slowed in the absence of HK. Thus, in a clotting assay dependent upon the formation of factor XIIa and the conversion of factor XI to factor XIa by factor XIIa, the absence of HK yields slow factor XII activation and virtually no activation of factor XI. Thus, HK deficiency [22,23] is almost as abnormal as factor XII deficiency if in vitro coagulation is employed as the assay. 

**Figure 5:**
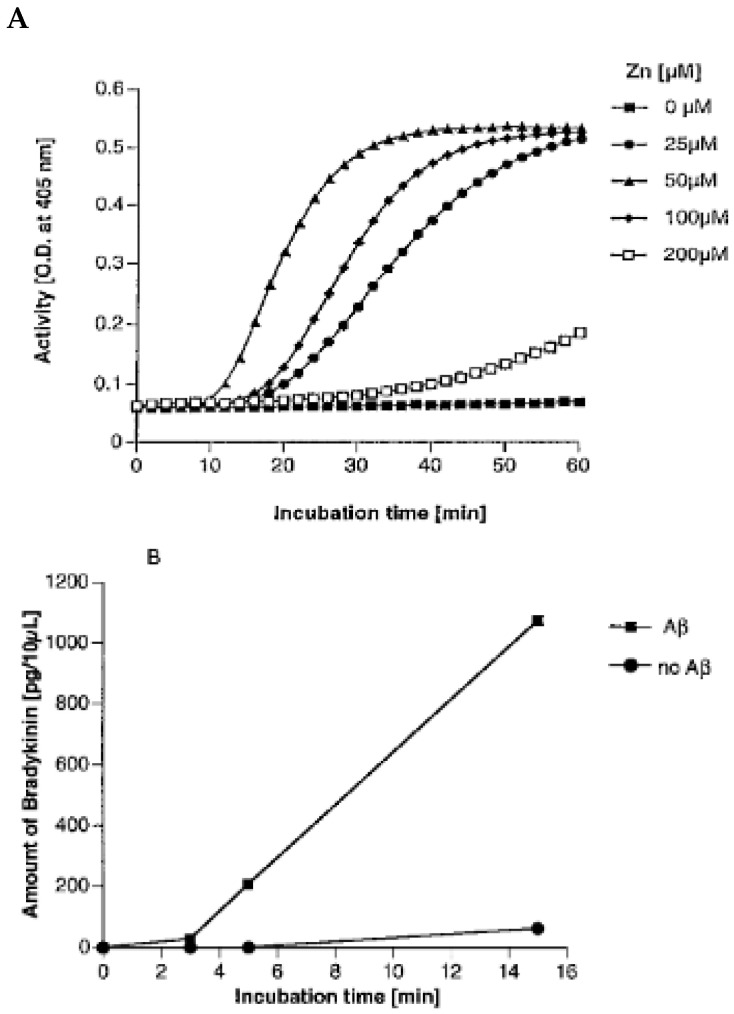
Activation by Aβ (1-42) and bradykinin formation. We have compared the rate of initiation of contact activation with aggregated Aβ (1-42) to that observed with aggregated Aβ (1-40) or Aβ (1-38), each at 50 µg/mL, upon incubation with factor XII (1 µg), prekallikrein (1 µg), HK (1 µg), 50 µM zinc, and 0.6 mM Chromozym PK. The rate of increase in prekallikrein to kallikrein is monitored at O.D. 405 nm (**A**). The rate of formation of bradykinin from the Aβ (1-42) mixture is shown in (**B**); a control containing no Aβ is included for comparison [6].

**Figure 6:**
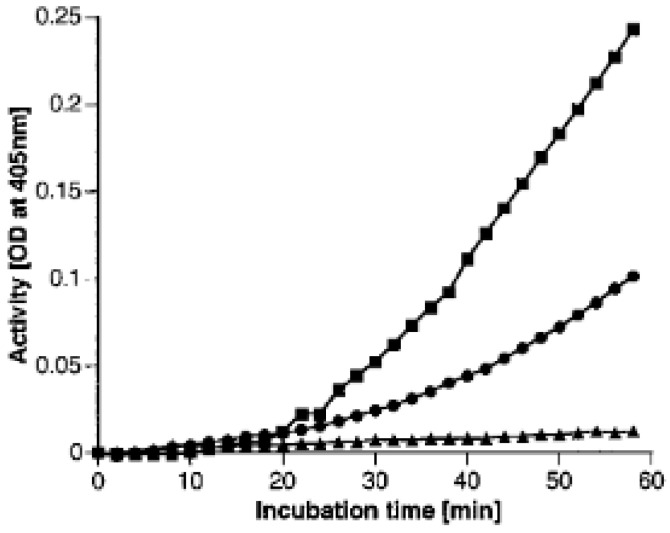
Role of factor XII and HK in the zinc-dependent activation of prekallikrein by Aβ (1-40). Five hundred micrograms/mL was preincubated for 3 days. Activation mixtures were then prepared with factor XII, prekallikrein, and HK (■); or factor XII and prekallikrein (●); or prekallikrein and HK (▲), each at 1 µg/mL, and incubated with 0.6 mM chromozym-PK and 50 µg/mL Aβ in the presence of 50 µM zinc ion. Conversion of prekallikrein to kallikrein O.D. 405 nm was monitored at 25 °C [6].

We also demonstrate the ability of aggregated Aβ to lead to bradykinin formation as the final step. This is depicted in Figure 5. At the top (Figure 5A), we compare the activation of the mixture of purified proteins comparing Aβ 1-38 (3-day incubation) with Aβ 1-40 (3 h incubation) with Aβ 1-42 (no incubation). Then, Figure 5B depicts the bradykinin evaluation as assessed by radioimmunoassay for the Aβ 1-42 protein. A prominent release is seen between 3 and 15 min, and the 15 min point represents the amount of bradykinin expected with the complete cleavage of the HK. The control at the bottom has no Aβ added. This was tested in the experiment depicted in Figure 6. Here, we employed Aβ 1-40 with a 3 h preincubation rather than Aβ 1-38 and allowed activation to proceed with a mixture of factor XII, prekallikrein, and HK; or factor XII and prekallikrein; or factor XII and HK. The uppermost curve contains all three proteins; the middle curve includes factor XII and prekallikrein, omitting the HK; and the flat line at the bottom omits factor XII and contains only HK and prekallikrein (i.e., the HK–prekallikrein complex). In the absence of factor XII, there is no activation. Thus, binding to initiate the autoactivation of factor XII by aggregated Aβ is the first step [6]. This was confirmed in separate experiments employing only aggregated Aβ plus factor XII, resulting in a slow autoactivation of factor XII, which was not observed in the absence of zinc ion [6]. The middle curve depicts the gradual activation of prekallikrein (by factor XIIa) when HK is absent. We do not distinguish the effect of HK on the rate of prekallikrein activation from the effect of HK on the kallikrein activation of factor XII. Clearly, HK is not an absolute requirement for the reciprocal activation of factor XII and prekallikrein as it is for factor XI.

**Figure 7:**
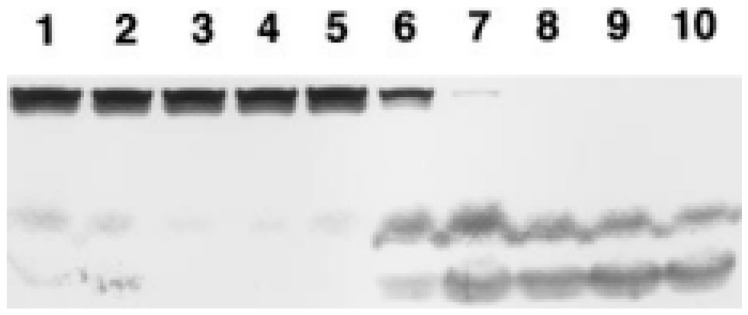
Immunoblot demonstrating cleavage of HK upon incubation of normal human plasma with aggregated Aβ (1-40) at 50 µg/mL compared to 1 µg/mL dextran sulfate. The final plasma dilution was 1:5 and sufficient zinc chloride was added for a final concentration of 50 µM. Lanes 1–4 represent HK upon incubation of plasma for 0 min, 30 min, 1 h, and 2 h in the absence of an added surface. Lanes 5–8 represent plasma incubated with aggregated Aβ at the same time intervals, and lanes 9 and 10 are 1 h and 2 h time points utilizing dextran sulfate [6].

The formation of bradykinin indicates that HK has been cleaved by plasma kallikrein. To assess HK cleavage, we switched to a plasma system in which aggregated Aβ 1-40 at 50 µg/mL was added and compared to 1 µg/mL dextran sulfate, a potent experimental activator of the kinin-forming cascade that is dependent on factor XII activation [25]. The final plasma dilution was 1:5 and sufficient zinc chloride was added for a final concentration of 50 mM. An SDS gel electrophoresis of HK is shown in Figure 7. Lanes 1–4 represent time points in the incubation of plasma for 0, 30 min, 1 h, and 2 h in the absence of any Aβ. Lanes 5–8 represent plasma incubated with the aggregated Aβ at the same time points. At 30 min, at least half the HK is cleaved and reaches 100% at 1 h. Lanes 9 and 10 are the 1 h and 2 h time points utilizing dextran sulfate as a positive control.

## 3. Activation of the Bradykinin Forming Pathway in Patients with Alzheimer’s Disease

While the ability of aggregated Aβ protein to activate the plasma bradykinin-forming pathway in vitro is clear, pathogenic significance requires (1) the demonstration of a similar activation of the kinin-forming pathway in patients, i.e., in vivo, and (2) a positive effect on disease progress by inhibiting this pathway, i.e., the arrest of mental decline in patients, or less likely, the reversal of symptoms. There is now abundant evidence (with a 15-year hiatus) that the aforementioned observations regarding the bradykinin-forming pathway are seen in patients with Alzheimer’s disease, with some evidence that activation is more readily observed in more severely affected subjects.

**Figure 8:**
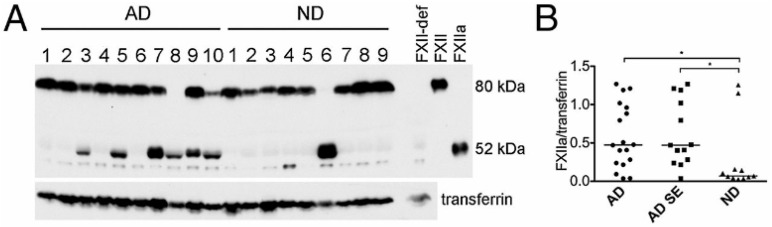
Activation of the FXIIa-driven contact system in AD patient plasma from Group 1. (**A**) Western blot analysis of FXIIa and transferrin loading control in plasma of 18 AD patients and 11 ND controls from Group 1 (representative samples shown), showing FXII zymogen (80 kDa) and the FXIIa heavy chain (52 kDa). The lane loaded with FXII-deficient human plasma (FXII-def) shows that the bands just below and above the FXIIa band are nonspecific. (**B**) FXIIa levels normalized to transferrin were significantly higher in AD patients (*p* = 0.029) than in ND plasma. When AD cases with a history of stroke (n = 5) were excluded from the analysis (AD SE), FXIIa levels remained significantly higher than in ND plasma (*p* = 0.018) [26]. * Clinically significant difference.

**Figure 9:**
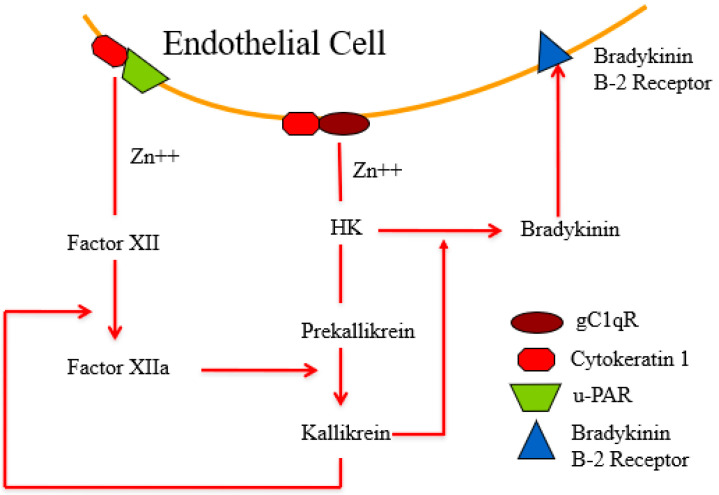
Zinc-dependent binding of factor XII and the HK–prekallikrein complex to endothelial cells. Factor XII is preferentially bound to the cytokeratin 1-uPAR complex, while HK is preferentially bound to gC1qR-cytokeratin 1. The latter can be a “two-hit” interaction with domains 5/6 binding to gC1qR (light chain) and domain 3 to cytokeratin 1 (heavy chain). Activation of factor XII to factor XIIa leads to conversion of prekallikrein to kallikrein. Kallikrein digests HK to liberate bradykinin which binds to the constitutively present B-2 receptor.

First, in vitro experiments by Zamolodchikov et al. reproduced our findings; factor XII was activated and HK was cleaved when the plasma of patients with Alzheimer’s disease was compared to normal controls [26]. While indirect, the pattern of a 52 Kd fragment of factor XII upon reduction is indicative of in vivo conversion to factor XIIa, while the cleavage of HK is directly proportional to the bradykinin generated [27,28] (Figure 8). Consistent with the latter observation is the presence of significant levels of plasma kallikrein in patients assessed with a chromogenic substrate [29]. Soon thereafter, with an improved method for the detection of cleaved HK, there was a positive correlation between the presence of dementia and neurolytic plaque scores [30]. The addition of an antibody to HK (in vitro) blocks the aggregated Aβ-induced cleavage of HK and the release of bradykinin [31], with the conclusion being that the antibody interferes with HK cleavage by plasma kallikrein. However, the elimination of HK from the kinin-forming cascade markedly reduces the activation of factor XII because HK catalyzes the reciprocal activation reactions of factor XII and prekallikrein [24]. This additional effect was not addressed [28,29,30,31], although a later study applying an antibody to a 20 amino acid region of HK domain 6 prevented HK binding of both prekallikrein and factor XI. In fact, the presence of this antibody caused the dissociation of both complexes from HK. Binding [32,33] is critical for intrinsic coagulation to proceed, and in vivo, the plasma proteins interact with binding sites on vascular endothelial cells [32,33,34,35,36] so that the initiation of the bradykinin-forming cascade may occur along the cell surface and is perpetuated and distributed in the fluid phase [37,38], as shown in Figure 9. As it stands, clinically, targeting factor XII/factor XIa or prekallikrein/kallikrein is more feasible than inhibiting the various functions of HK.

**Figure 10:**
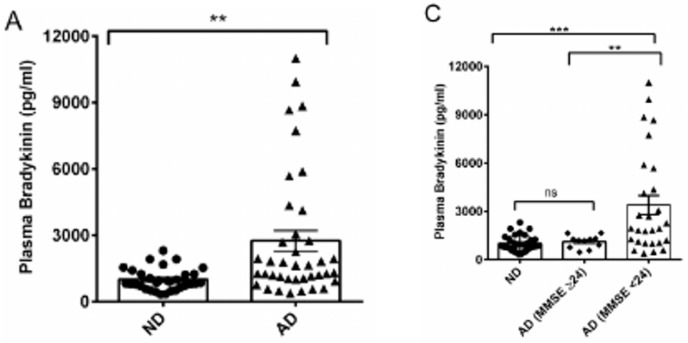
Plasma bradykinin level in ND and AD patients. (**A**) Plasma bradykinin levels from ND (n = 32) and AD (n = 39) subjects were quantified by ELISA. Plasma bradykinin was significantly higher in AD than in ND samples. (**C**) AD samples were grouped according to their mini-mental state examination (MMSE) scores (MMSE ≥ 24), mild dementia, Group 1; MMSE < 24, moderate to severe dementia, Group 2, and their bradykinin levels were compared. Group 2 individuals presented with significantly higher bradykinin compared to ND and Group 1 subjects. ND = normal; AD = Alzheimer’s Disease [39]. **, *** Level of clinical significance.

**Figure 11:**
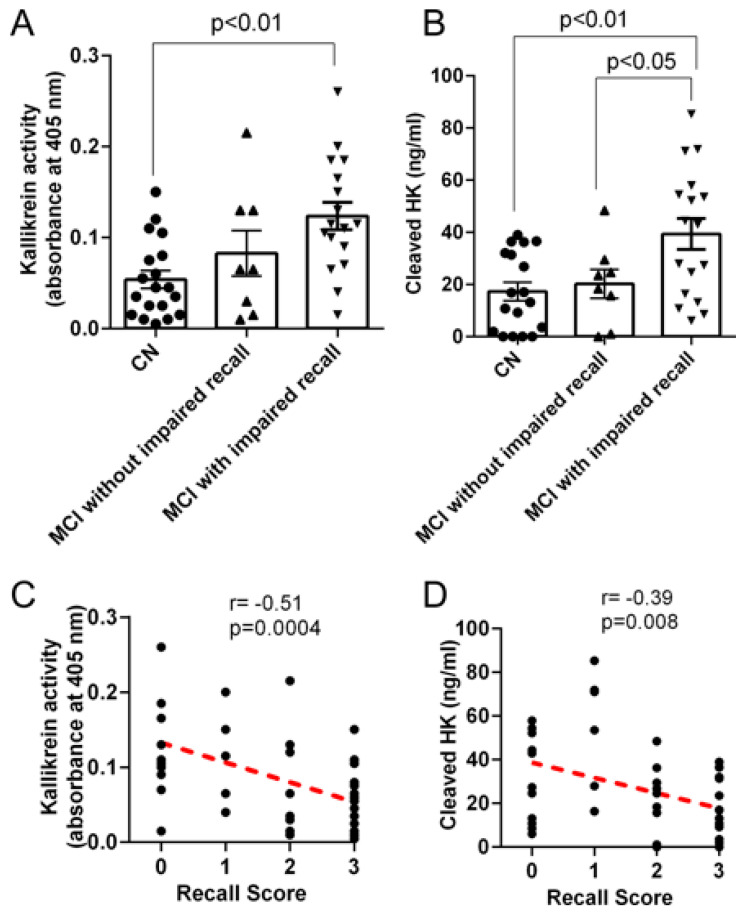
Plasma kallikrein activity and cleaved HK levels in plasma from minimal cognitive improvement (MCI) and cognitively normal (CN) subjects were assessed using chromogenic assay and ELISA, respectively. MCI patients with low recall scores (0 or 1) were grouped as “MCI with impaired recall”. MCI patients with higher recall scores (2 or 3) were grouped as “MCI without impaired recall”. (**A**) Plasma kallikrein activity was significantly higher in MCI patients with impaired recall memory compared to that of CN. (**B**) Plasma cleaved HK levels were significantly higher in MCI patients with impaired recall compared to CN. (**C**) Plasma kallikrein activity inversely correlates with recall score. (**D**) Plasma cleaved HK level also inversely correlates with recall score. Statistical analysis was performed by one-way ANOVA followed by Tukey’s multiple comparison test. Correlation was analyzed using Pearson’s correlation coefficient (r). Results are presented as mean ± SEM. n = 19 CN, 25 MCI. The difference in cleaved HK [29].

The blockade of particular components of the bradykinin-forming cascade and bradykinin itself has had ameliorating effects on the progression of Alzheimer’s disease. Blood levels of plasma kallikrein, cleaved HK, and bradykinin levels were all significantly elevated when patients’ cognitive changes were followed (Figure 10 and Figure 11). The values exceeded those obtained with patients in whom Alzheimer’s disease was diagnosed but memory was still intact. The correlation coefficients for recall scores were 0.39 and 0.51 for HK and kallikrein, respectively (Figure 11). Thus, further studies with larger numbers of patients would be desirable. These latter values, although modest, were still elevated compared to normal controls. In addition, elevated plasma bradykinin levels were the most prominent in patients with severe disease [39]. In post-mortem studies, bradykinin interacted with and/or colocalizes with “fibrillar” Aβ and Aβ plaques.

More recent studies, in which plasma and cerebrospinal fluid (CSF) of patients were assessed, confirmed findings of increased plasma levels of factor XIIa and plasma kallikrein in patients. Further, the magnitude of the plasma concentration of each enzyme correlated with the stage of Alzheimer’s dementia, from mild prodromal presentations to severe disease. Factor XIIa activity could serve as a diagnostic biomarker [26], as can cleaved HK [30]; the CSF ratio of Aβ (1-42)/plasma factor XIIa could be added to yield improved accuracy when employed together. Lastly, as a consequence of the role of factor XII activation due to binding to aggregated Aβ, there are increased levels of factor XI in the plasma of patients, which are also related to diminished cognitive function [40], and the activation of the coagulation cascade proceeds to thrombin formation [41] even if clinical thrombosis is not seen.

## 4. Discussion

Among the earliest observations regarding the bradykinin-forming cascade in Alzheimer’s disease include the presence of factor XII (Hageman factor) bound to the plaques present in the brain of Alzheimer’s patients [42] and the presence of cleaved HK in the cerebrospinal fluid of patients [30,43]. Bradykinin, once released, promotes inflammatory processes including increased blood–brain barrier permeability, edema, and cytokine expression [44]. Further, any local injury or inflammation within the brain might release tissue kallikrein, which can cause local bradykinin formation and recruit the plasma proteins secondarily. These observations suggest communication between plasma proteins, the brain parenchyma, and spinal fluid. The effects of bradykinin as a vasodilator and to increase vascular permeability could facilitate such equilibria between compartments and also allow monoclonal antibodies to bind and diminish deposits of Aβ (i.e., current therapy), given subcutaneously or intravenously, and migrate from plasma to the brain. The idea that aggregated Aβ is a potent activator of the bradykinin-forming cascade [6] was later generalized [7] to encompass a diverse array of aggregates or misfolded proteins such as cryoglobulins.

An important multifaceted role for zinc has been identified. It greatly facilitates the formation of plaques [45] and has a role in the secretion of the Aβ precursor to produce Aβ peptides [46]. Our observations demonstrate an important role for zinc in producing Aβ aggregates and activating the bradykinin-forming cascade, employing such aggregates as a “surface”. Although factor XII possesses very low intrinsic activity (i.e., not zero), binding to initiating surfaces renders factor XII cleavable [47] with the activation to two molecular forms by either plasma kallikrein or plasmin [48]. These include factor XIIa (or α factor XIIa, molecular weight 80 Kd) and secondarily, factor XII fragment (factor XIIf or β factor XIIa; molecular weight 30 Kd) [21,49]. A role for zinc ion has also been observed in the interaction of factor XII and the HK–prekallikrein complex with cell surfaces such as endothelial cells [33,50]. The optimal zinc concentration is between 15 and 50 µM, which is the same as the concentration reported to accelerate Aβ aggregation [45]. Endothelial cells have specific cell membrane proteins with which interaction takes place [51], including gC1qR, cytokeratin 1, u-PAR [urokinase plasminogen activation receptor], and to a lesser degree, sulfated proteoglycans [52,53].

In plasma, one can readily demonstrate the activation of all steps from factor XII activation to bradykinin formation in the presence of chelating agents to prevent peptide degradation. No ion is requisite (in plasma) for bradykinin formation, yet zinc does, apparently, affect the kinetics of plasma activation, i.e., it is somewhat slower if zinc is absent [54,55,56]. Zinc binds to factor XII [54] and domain 5 of HK, which may account for the observation, at least in part [57].

The effects of the production of activated factor XII, kallikrein, bradykinin, and cleaved HK upon disease pathogenesis have not yet been determined. To determine the effects, blocking the pathway in patients is required. Drugs that could be employed are now standard treatment (prophylaxis) for hereditary angioedema types I and II (C1 inhibitor deficiency). The monoclonal antibody to kallikrein (lanadelumab) would be of particular interest [58] and is administered subcutaneously. A new antibody with the same specificity but modified to last 3 months with a single injection is currently being studied [59]. A monoclonal antibody to factor XIIa is also at the final steps needed for approval [60]. Icatibant, which blocks bradykinin at the B_2_ receptor level lasts only a few hours, but its half-life can also be increased markedly [61], with potential for prophylactic use.

## 5. Additional Considerations

There are many possible consequences of bradykinin formation within the central nervous system, and the plasma proteins required for bradykinin formation may independently modulate the function of nerve cells, glial cells, or vascular endothelial cells; the consequences of this are not yet clear. We also do not know the cause of the initial disruption of the blood–brain barrier to facilitate migration to and from the central nervous system. One possibility is that bradykinin could facilitate the migration of plasma proteins into the sites of Aβ aggregation, including proteins of the complement cascade [62]. There is evidence of complement activation in Alzheimer’s disease [63] as well. Bradykinin, plasma kallikrein, and activated factor XII all have interactions with endothelial cells, astrocytes of the central nervous system, and smooth muscle cells [64,65] that might not only facilitate local activation via aggregated Aβ but might also affect the cellular production of Aβ [66]. An increase in the plasma level of factor Xa as Alzheimer’s disease progresses indicates that the coagulation cascade beyond factor XIIa or factor XIa is activated even if thrombosis does not occur. Enzymatic activity and bradykinin levels in the blood increase as Alzheimer’s dementia progresses, while the level of Aβ 1-42 and bradykinin in cerebrospinal fluid actually decreases [67]. 

There remains a possibility that the binding of proteins such as factor XII and HK to cells within the central nervous system may serve to activate them. Factor XII appears to be a mitogen via its epidermal growth factor domains and does so via the activation of an intracellular mitogen-activated protein kinase [64]. The enzyme kallikrein is a chemotactic factor and an activator of neutrophils and monocytes [68,69]. Cleaved HK induces the apoptosis of endothelial cells and increases the presence of reactive oxygen species [65], including nitric oxide [66]. The two functions of cleaved kininogen not present in the native protein include the induction of endothelial cell apoptosis and the inhibition of angiogenesis [65,67]. These functions are dependent on domain 5 [67,70], which possesses the surface binding site and the site for zinc interaction [57]. Domain 5 of HK mediates interactions with leukocytes, which result in the inhibition of adhesion to endothelial cells so that cell recruitment is impeded [71]. The inhibition of angiogenesis has been shown to result from cleaved HK interacting with endothelial cell tropomyosin [72]. Thus, the possible effects of cleaved HK are multifaceted because cleaved HK, upon binding to endothelial cells, also causes the release of cytokines [73] and chemokines; thus, it is simultaneously proinflammatory and anti-inflammatory. Further, the binding of HK and factor XII to cells such as vascular smooth muscle cells activates MAP kinase P44/42. Thus, the intracellular effects of binding to endothelial cells could be important for the progression of Alzheimer’s disease apart from the consequences of bradykinin production. Similar studies employing neurons and glial cells may shed light on the various effects of the constituents of the bradykinin-forming cascade.

## 6. Concluding Comment

It is clear that aggregated Aβ protein is a potent stimulus for the activation of all steps of the plasma bradykinin cascade and is demonstrable in patients. Some parameters measured reflect the severity/stage of the disease. With multiple drugs that inhibit plasma kallikrein or activated factor XII, one can proceed with controlled therapeutic trials to determine whether this inhibition arrests the progress of the disease or even improves cognition.

## Data Availability

Not applicable.
